# Primary squamous cell carcinoma of the breast: a case report

**DOI:** 10.1007/s12672-024-00958-6

**Published:** 2024-04-01

**Authors:** Xibo Liu, Jiahui Chen, Chuanling Hou

**Affiliations:** Department of Pathology, Shaoxing People’s Hospital, No. 568, Zhongxing North Road, Shaoxing, 312000 Zhejiang China

**Keywords:** Squamous cell carcinoma, Spindle cell, Breast, Pathology, Case report

## Abstract

**Introduction:**

Squamous cell carcinoma (SCC) of the breast is a rare variant of invasive breast cancer that has been classified as metaplastic carcinoma. When a tumor is composed of spindle cells, diagnosis is challenging.

**Case report:**

A 42-year-old woman with a large mass in the right breast underwent modified radical mastectomy. A pathological examination revealed a tumor with central necrosis in it. The tumor had a sarcomatoid growth pattern and the cells were spindle-shaped with severe atypicality. Immunohistochemical staining showed that P63, P53, vimentin, and CKpan were positive, whereas estrogen receptor and C-erbB-2 were negative. Ki-67 proliferation index was as high as 90%. Therefore, a diagnosis of SCC of the right breast was made. The patient received eight cycles of postoperative chemotherapy with paclitaxel and carboplatin, followed by seven cycles of radiotherapy. During follow-up, the patient also had a left thyroid tumor, and postoperative pathology suggested microinvasive follicular carcinoma. Since breast surgery, the patient has remained disease-free for more than four years.

**Conclusion:**

SCC of the breast with spindle cell and sarcomatoid features is rare. The diagnosis of such tumors requires exclusion of tumors with similar histological morphologies.

## Introduction

Metaplastic carcinomas are a group of invasive breast cancers with diverse characteristics. These cancers are characterized by the transformation of neoplastic epithelial cells into squamous or mesenchymal cell types such as spindle, chondroid, and osseous cells. Metaplastic carcinomas account for approximately 0.2—1% of all invasive breast cancers [1]. Squamous cell carcinoma (SCC) of the breast is a rare type of metaplastic cancer that accounts for approximately 0.1% of all invasive malignancies [2]. Owing to its extremely low prevalence, there is limited knowledge about its clinical presentation and lack of standardized treatment for SCC of the breast [3]. The prevalence of breast SCC has increased in recent years, possibly as a result of improved diagnostic tools and physicians' heightened awareness of the various types of breast cancer and their influence on prognosis [4]. Typical SCC can easily be identified by histological examination. However, accurate pathological diagnosis may be difficult when tumor differentiation is poor. This report focuses on a case of SCC of the breast with sarcomatoid histological features.

## Case report

A 42-year-old female patient reported a mass in her right breast that had been present for a year. One year prior, she noticed an egg-sized painless mass in her right breast. The patient did not have any serious concerns or seek treatment. Despite initial indifference, the tumor continued to grow. Two months prior, the patient observed a significant increase in mass, approximately one fist size. Slight tenderness associated with the mass was observed. Therefore, the patient visited our hospital for treatment. She had experienced ulcerative colitis three years ago which was alleviated after treatment with mesalazine and bifid triple viable enteric-coated capsules.

Physical examination revealed an 8 × 7 cm mass located in the patient 's right breast. The mass was hard, mobile, tender, and circumscribed. No depression, ulceration or orange peel-like changes were found in the skin. No obvious masses were palpable on the left breast. No nipple discharge was observed on either side. The axillary and supraclavicular lymph nodes were unswollen.

**Fig. 1:**
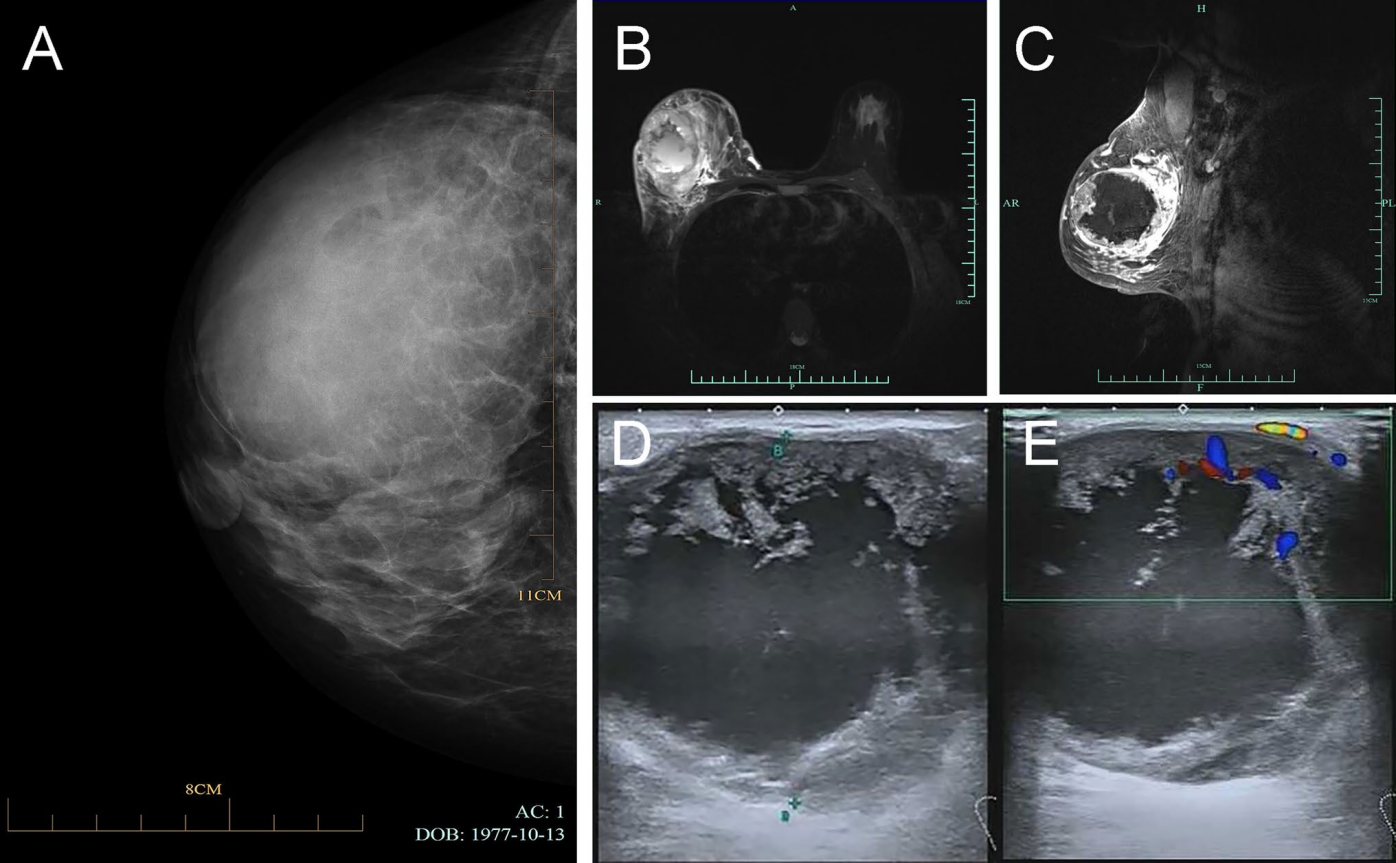
The imaging analysis of breast SCC in the upper outer quadrant of the right breast is presented. Mammography revealed a slightly elevated oval mass with a clear margin and homogeneous internal density **A**. The MR and DWI examinations (3.0 T) depicted a mass measuring 85 × 57 × 82 mm in size in the lateral quadrant of the right breast, invading surrounding tissues **B**, **C**. Ultrasonography revealed a 73 × 53 mm cystic-solid nodule with well-defined borders and a regular shape **D**, while blood flow signals were observed in the solid portion **E**

**Fig. 2:**
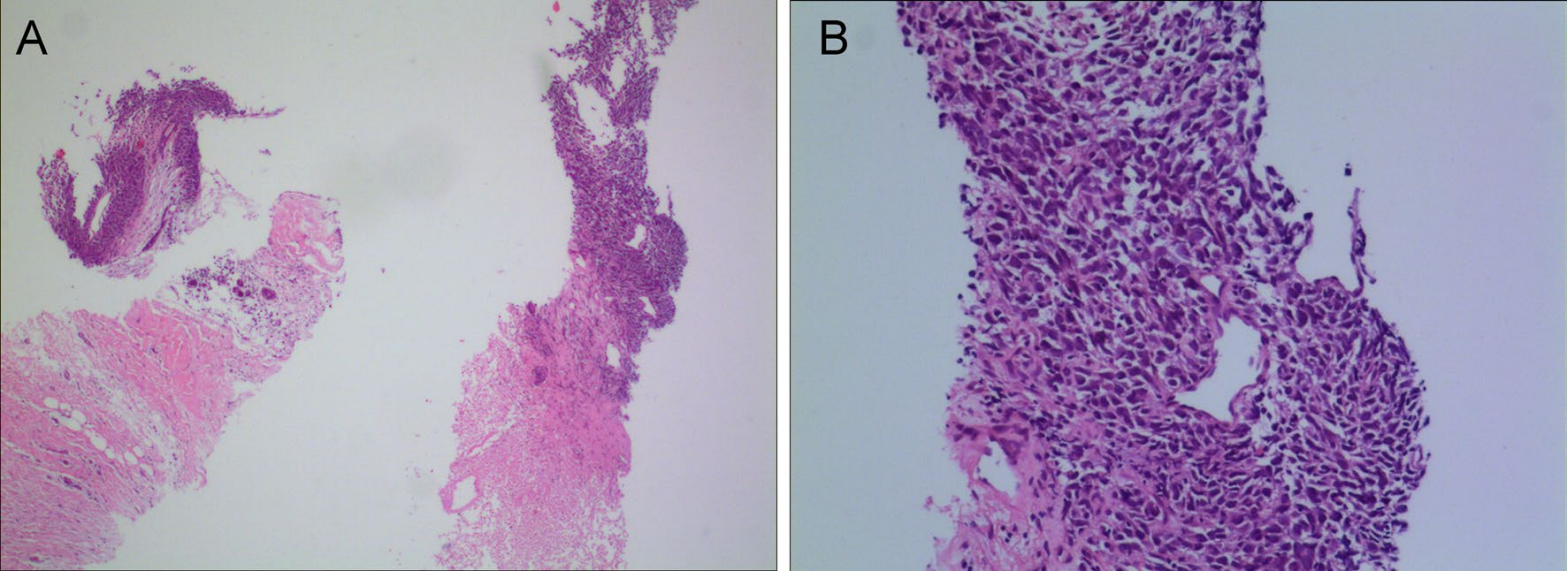
Images of breast cancer tumor biopsy samples. **A** H&E staining of a tumor showing necrosis at 40 × magnification. **B** Tumor cells appear irregular and atypical in shape, as observed under 200 × magnification using H&E staining

Laboratory tests revealed elevated levels of CA125 at 45.1 U/ml, LDH at 442.8 U/L, PRL at 1534.09 mIU/L, and TSH at 7.4058 µIU/ml, respectively. Mammography revealed a mass in the right breast, measuring 93 × 76 mm, with a clear boundary and uniform inner density. Enhanced MR imaging and diffusion-weighted imaging (DWI) on a 3.0 T scanner revealed an 85 × 57 × 82 mm tumor with uneven signals, predominantly low signals on T1-weighted images, and high signals on T2-weighted images. Diffusion was limited. After enhancement, the mass showed continuous enhancement with annular enhancement in the arterial phase (Breast Imaging Reporting and BI-RADS: 4 B). Ultrasonography of the breast revealed a solid cystic nodule measuring 73 × 53 mm with a clear boundary and a regular shape in the upper outer quadrant of the right breast. The blood flow signals were visible in the solid portion (Fig. 1). Ultrasonography of the thyroid gland showed an echogenic nodule measuring 9 × 6 mm in the lower part of the right lobe, with a distinct border and a regular shape. Additionally, a 30 × 20 mm hyperechoic nodule with a clear boundary and regular shape was observed in the lower part of the left lobe. No enlarged lymph nodes were detected on either side. An ultrasound scan of other organs, including the liver, gallbladder, pancreas, and spleen, revealed no abnormalities. An ultrasound-guided breast biopsy was performed to determine the nature of the breast mass. Pathological examination of the biopsy samples indicated a poorly differentiated malignant tumor with necrosis (Fig. 2). One week later, the patient underwent modified radical mastectomy for right breast cancer under general anesthesia.

**Fig. 3:**
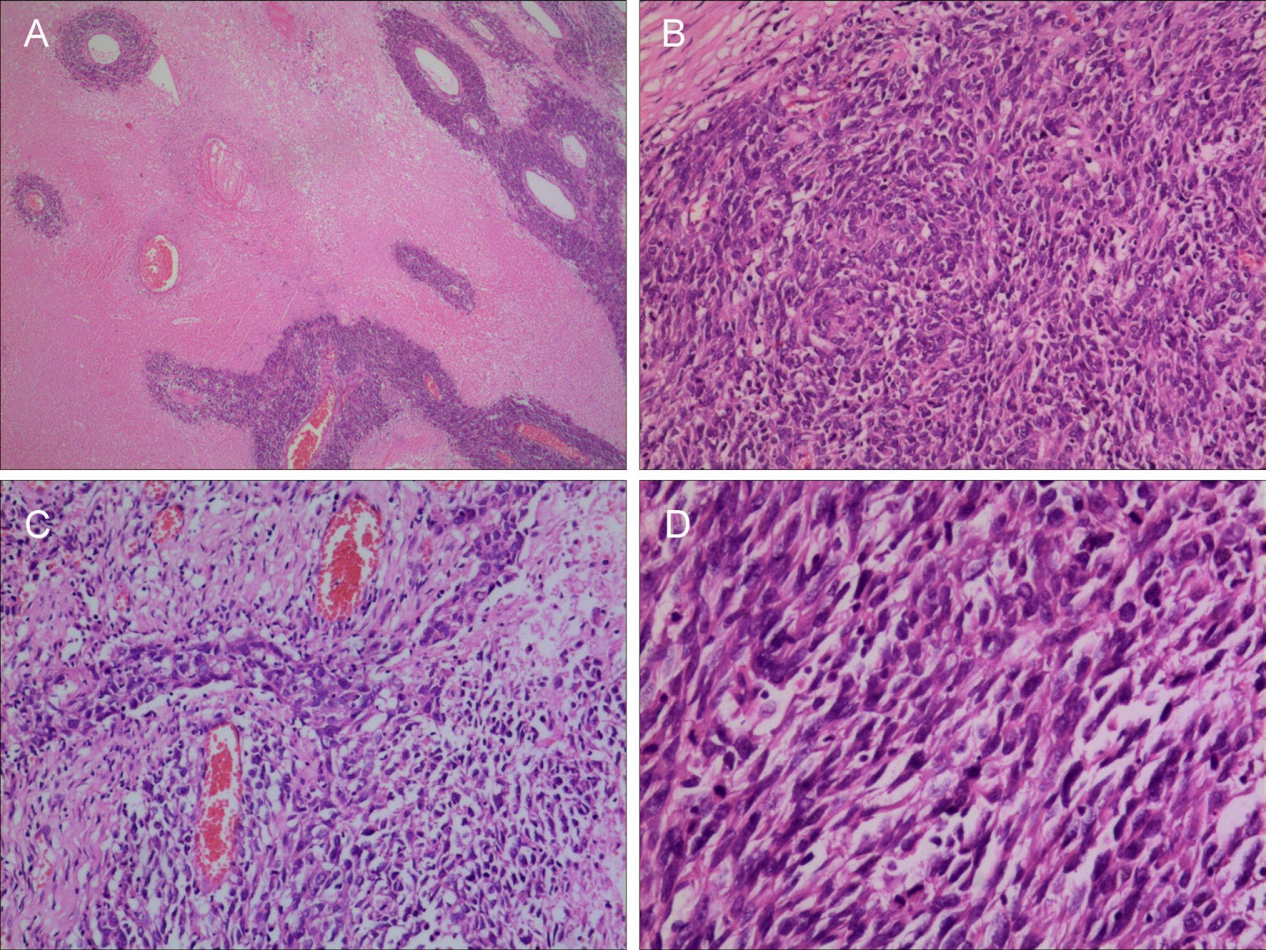
Microscopic examination of breast SCC (H&E staining). **A** A large area of coagulative necrosis was seen in the center of the tumor tissue, and residual tumor cells could be observed around the blood vessels. **B** Tumors composed of malignant spindle cells were visible. **C** Local tumor cells formed a solid nest structure similar to the morphological characteristics of ductal carcinoma. **D** The tumor cells were spindle-shaped, diffusely distributed, and arranged in bundles or sheets. Atypia of the tumor cells was evident with large nuclei, deeply stained chromatin, more mitotic figures, and no visible nucleoli. (Amplification: A, × 40, B and C, × 100, D, × 200)

**Fig. 4:**
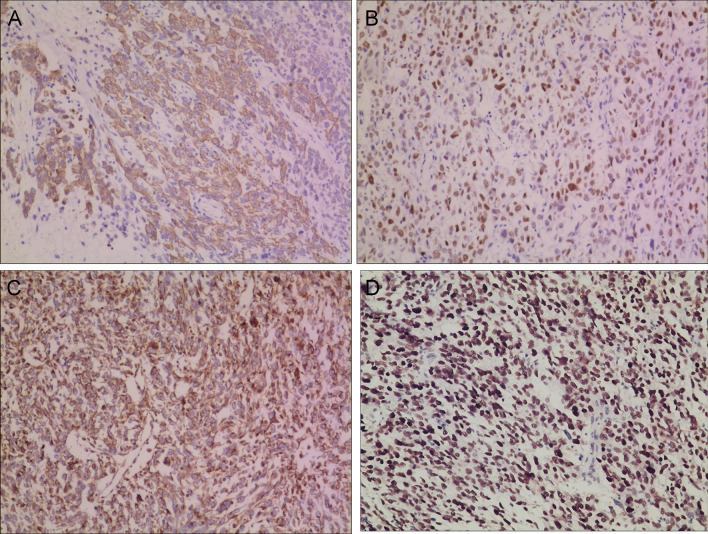
Immunohistochemical analysis of the tumor reveals reactivity for CKpan **A**, P63 **B**, Vimentin **C**, and a high Ki-67 proliferation index **D**. (Amplification: × 100)

Grossly, the size of the tumor was 6 × 4.5 × 3.3 cm, most of which were cystic and contained necrosis. Microscopic examination of surgical specimens revealed that the tumor was predominantly composed of sarcomatoid spindle cells with severe atypia. A wide range of necrosis was observed in the central region of the tumor. The remaining tumor cells were observed in the necrotic area, with these cells tending to grow around the blood vessels. Tumor cells far from the vascular area formed a large area of coagulation necrosis. The tumor cells were diffusely distributed, arranged in bundles and flakes, disorderly, and locally woven. Atypia of the tumor cells was evident with large nuclei, deeply stained chromatin, more mitotic figures and no visible nucleoli. Only a few tumor cells were arranged in solid strip shapes, similar to the morphological characteristics of ductal carcinoma (Fig. 3). Immunohistochemical staining revealed positivity for P63, Vimentin, P53, and CKpan, whereas CK8/18, CD34, desmin, S-100, SMA, HMB-45, Melan-A, and estrogen receptor were negative. There was no overexpression of CerbB-2. Approximately 3% of the tumor cells exhibited weak-to-moderate progesterone receptor expression. The Ki-67 proliferation index was approximately 90% (Fig. 4). The pathological diagnosis was non-keratinizing SCC of the right breast with a negative margin. No tumor metastasis was observed in 46 right axillary lymph nodes or six right subclavian lymph nodes. The patient underwent gynecological and otorhinolaryngological examinations, and no tumors were found in the cervix or nasopharynx.

**Fig. 5:**
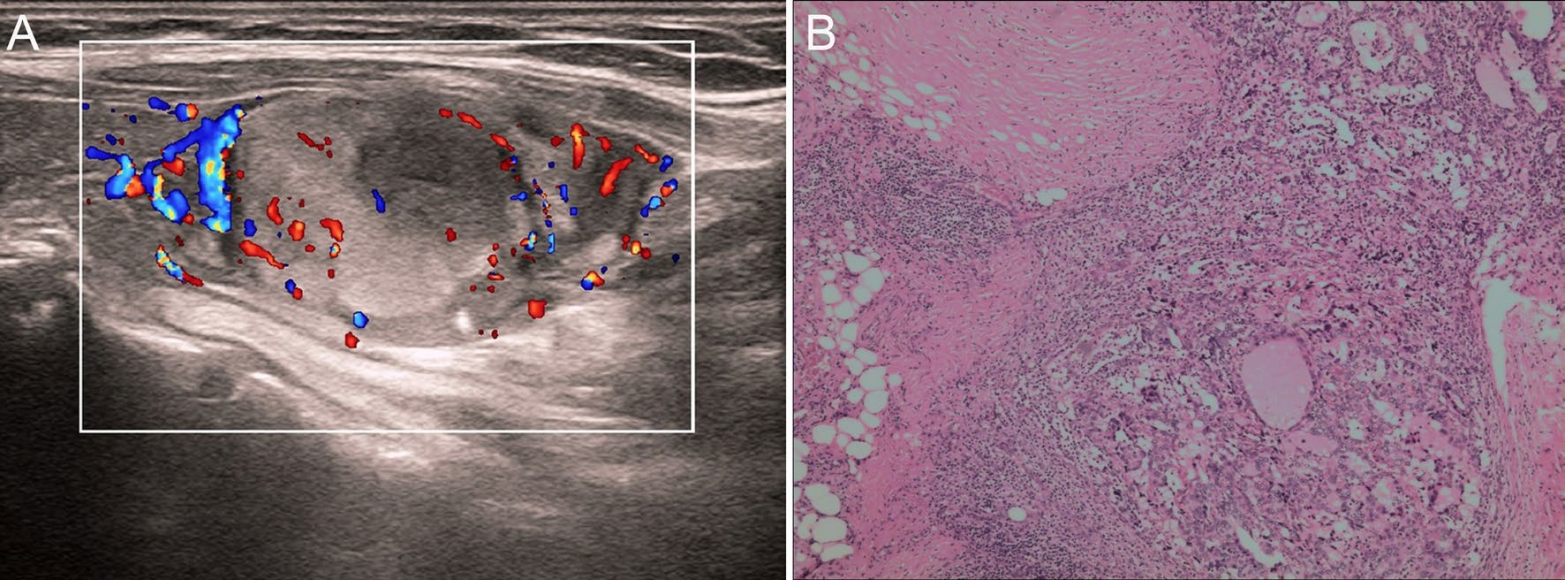
Ultrasound and pathological examination of thyroid tumors. **A** An ultrasound examination revealed a hypoechoic nodule, measuring 36 × 17 x 21 mm, situated in the left lower pole of the thyroid gland. It exhibited well-defined borders and regular structure. The nodule was filled with intense blood flow signals. **B** The pathological examination indicated a microinvasive follicular carcinoma of the thyroid. Tumor cells penetrated the thick fibrous capsule. (Amplification: × 40)

After surgery, the patient underwent chemotherapy with a combination of docetaxel 115 mg and carboplatin 550 mg for eight cycles. The patient subsequently received seven cycles of radiotherapy (30 Gy/15 F 50 Gy/25 F). Three years after the operation, ultrasound examination revealed a 4 × 4 mm hypoechoic nodule in the middle of the right thyroid gland and a 36 × 17 × 21 mm mixed echoic nodule in the lower pole of the left gland, which were larger than before (Fig. 5A). The thyroglobulin antibody level was as high as 281.83 IU/mL, while the anti-thyroid peroxidase antibody level was as high as 968.37 IU/mL. The patient was re-hospitalized for surgical treatment, and the left substernal and right parts of the thyroid gland were removed. Postoperative pathological examination revealed a microinvasive follicular carcinoma in the left thyroid (Fig. 5B) and an adenomatous hyperplastic nodule in the right thyroid. Twenty months after thyroid surgery, the patient came to the hospital for re-examination, and no evidence of breast or thyroid tumor recurrence was found. She had been treated with gayoule tablets for hypothyroidism for a long time, and no adverse reactions were observed during treatment. The patients were satisfied with the treatment and care.

## Discussion

The etiology of SCC of the breast remains unclear [5]. It has been reported to develop in patients undergoing breast implant augmentation [6, 7]. Additionally, there are reports suggesting a possible association between SCC of the breast and Zuska's disease [8]. SCC of the breast has been discovered in females aged 29–90 years, with no reported instances in males [9]. SCC of the breast is a histological subtype of metaplastic carcinoma with similar clinical features to non-specific ER-negative invasive breast cancer [1], despite reports of HER2 overexpression [10, 11]. Most cases present as palpable masses and are identified as mass lesions on ultrasonography or mammography [12]. Additional symptoms besides breast lumps, such as skin ulcers and fungal infections can affect the chest wall [4].

In this case, the abnormal structure mostly consisted of spindle cells displaying a sarcomatous growth pattern, which lacks the characteristic histological features of SCC, such as keratinization and intercellular connections. This presents a considerable difficulty in arriving at a definitive pathological diagnosis. Differential diagnoses mainly include spindle cell carcinoma and phyllodes tumors of the breast. Spindle cell carcinoma is another histological subtype of metaplastic carcinoma. These tumor cells can form small clusters of areas with more epithelioid morphology or squamous differentiation. This group of tumors includes lesions that may constitute the spectral end of spindle squamous cell carcinomas [1]. Although both SCC and spindle cell carcinoma can express P63, positive expression of P53 is more likely in SCC. Malignant phyllodes tumors typically exhibit sarcomatoid patterns in stromal components. Its histological structure is leaf-like and benign glandular epithelium, with obvious proliferation of mesenchymal cells, obvious cell atypia, and frequent mitotic figures. In our case, although the specimens were thoroughly inspected, the absence of glandular structure offered evidence to exclude phyllodes tumors. Malignant phyllodes tumors do not express epithelial markers and are mostly negative for P63. In addition, the diagnosis of breast primary non-keratinizing SCC also needs to rule out the possibility of metastasis from other organs such as the cervix [13].

Owing to the rarity of this tumor, there are no common guidelines for its treatment. Apart from surgery, postoperative adjuvant therapy for metaplastic carcinoma is similar to that for invasive ductal carcinoma, but there are insufficient data on its efficacy [14]. Thus, the standard treatment regimens for ductal carcinoma may not be effective for breast SCC. In a case report, a patient who received radiation therapy and adjuvant chemotherapy developed recurrent disease and eventually died [15]*.* Therefore, multidisciplinary treatment is beneficial for patients in complex individual situations [16]. One-year and five-year etiology-specific survival rates for breast SCC are 81.6% and 63.5%, respectively [17]. A retrospective study demonstrated that individuals with breast SCC had an average disease-free survival rate of 24 months and an overall survival rate of 40 months [3].

Our patient was satisfied with the results of treatment and care, although she also underwent thyroid cancer surgery after breast surgery, postoperative chemotherapy, and radiotherapy. At present, she is in good health and has survived for more than four years without tumors after breast tumor surgery.

In summary, for the first time, we have provided a detailed description of the clinical and pathological features of a case of breast SCC with spindle cell and sarcomatoid features. This case posed a certain challenge for pathologists, as spindle cell carcinoma and phyllodes tumors must be excluded when making pathological diagnoses.

## Data Availability

The datasets generated during and/or analyzed during the current study are available from the corresponding author on reasonable request.
